# Australian Fur Seals (*Arctocephalus pusillus doriferus*) Use Raptorial Biting and Suction Feeding When Targeting Prey in Different Foraging Scenarios

**DOI:** 10.1371/journal.pone.0112521

**Published:** 2014-11-12

**Authors:** David P. Hocking, Marcia Salverson, Erich M. G. Fitzgerald, Alistair R. Evans

**Affiliations:** 1 School of Biological Sciences, Monash University, Melbourne, Victoria, Australia; 2 Geosciences, Museum Victoria, Melbourne, Victoria, Australia; 3 Wild Sea Precinct, Zoos Victoria, Melbourne, Victoria, Australia; New York Institute of Technology College of Osteopathic Medicine, United States of America

## Abstract

Foraging behaviours used by two female Australian fur seals (*Arctocephalus pusillus doriferus*) were documented during controlled feeding trials. During these trials the seals were presented with prey either free-floating in open water or concealed within a mobile ball or a static box feeding device. When targeting free-floating prey both subjects primarily used raptorial biting in combination with suction, which was used to draw prey to within range of the teeth. When targeting prey concealed within either the mobile or static feeding device, the seals were able to use suction to draw out prey items that could not be reached by biting. Suction was followed by lateral water expulsion, where water drawn into the mouth along with the prey item was purged via the sides of the mouth. Vibrissae were used to explore the surface of the feeding devices, especially when locating the openings in which the prey items had been hidden. The mobile ball device was also manipulated by pushing it with the muzzle to knock out concealed prey, which was not possible when using the static feeding device. To knock prey out of this static device one seal used targeted bubble blowing, where a focused stream of bubbles was blown out of the nose into the openings in the device. Once captured in the jaws, prey items were manipulated and re-oriented using further mouth movements or chews so that they could be swallowed head first. While most items were swallowed whole underwater, some were instead taken to the surface and held in the teeth, while being vigorously shaken to break them into smaller pieces before swallowing. The behavioural flexibility displayed by Australian fur seals likely assists in capturing and consuming the extremely wide range of prey types that are targeted in the wild, during both benthic and epipelagic foraging.

## Introduction

Raptorial biting or pierce feeding was traditionally thought to be the primary prey capture tactic used by most pinnipeds when hunting underwater [1], [2]. This hunting tactic involves actively pursuing prey, before striking out with the head or accelerating the whole body to seize prey in the teeth and jaws by biting or snapping [3]. This has been suggested to be the original feeding mode in pinnipeds, as it requires few changes from the original terrestrial bauplan [3]. This is supported by observed tooth condition in some of the earliest fossil pinnipeds, including *Enaliarctos* and *Pteronarctos*, which were found to have distinct wear facets on their teeth, indicating their use in piercing and cutting food [1]. However, study of the feeding mode in extant phocid seals has shown a number of species to instead use suction feeding for prey capture underwater, including the crabeater seal (*Lobodon carcinophaga*) [4], bearded seal (*Erignathus barbatus*) [5], leopard seal (*Hydrurga leptonyx*) [6] and harbour seal (*Phoca vitulina*) [7]. Among extant pinnipeds, the otariid seals (fur seals and sea lions) are often considered to perform raptorial feeding rather than suction feeding [7]; however, no detailed description has been published documenting their foraging behaviours from first-hand observations.

In this present study we therefore sought to test the hypothesis that otariid seals primarily perform simple raptorial biting when capturing prey underwater. To do this we performed controlled feeding trials with captive Australian fur seals (*Arctocephalus pusillus doriferus*). When hunting at sea this species consumes a wide range of prey, including both schooling epipelagic prey and benthic fish and cephalopods [8]–[11]. In one study, prey DNA collected from scat samples represented 54 species of bony fish, four cartilaginous fish and four species of cephalopod [12]. Australian fur seals are unusual amongst fur seals in that they primarily perform benthic foraging near the seafloor over the continental shelf, rather than epipelagic foraging in open water, which is more typical of other fur seal species [13]–[15]. This has been identified though use of time-depth recorders that show them to perform U-shaped dives, where the seal descends directly to the seafloor before tracking along the bottom in search of prey [16]. Arnould and Hindell [16] found that 78% of foraging dives performed by Australian fur seals hunting in central Bass Strait, between Tasmania and mainland Australia, were U-shaped dives close to the seafloor, while the remaining foraging dives were V-shaped dives associated with epipelagic foraging. Given the different conditions faced when hunting near the seafloor versus in open water, it is therefore possible that Australian fur seals may vary their prey capture behaviours when hunting in different settings. Hence, it was also the aim of this study to explore the range of foraging behaviours displayed by captive fur seals when encountering prey that has been presented in different ways.

## Methods

### Study Animals

Detailed observations were made during feeding trials carried out in the main seal pool in the ‘Wild Sea’ precinct of Melbourne Zoo (Elliott Ave, Parkville 3052, VIC, Australia). Two adult female Australian fur seals (Bay and Tarwin) were observed in this study (Table 1). Both were brought into captivity after being rescued from the wild in poor condition as juveniles and being deemed unsuitable for release. They lived on display as part of the permanent collection at Melbourne Zoo, where they had also been trained using positive reinforcement to take part in educational displays for the zoo's visiting public. All observations and protocols carried out as part of this work were done under the approval of the Zoos Victoria Animal Care and Ethics Committee (ZV12007; ZV12012).

**Table 1:** Experimental subjects.

Subject	ARKS #	Species	Sex	Est. Date of birth	Body Length (cm)	Girth (cm)	Mass (kg)
Bay	A70598	**Australian fur seal** *Arctocephalus pusillus doriferus*	F	November 2006	136	88	48
Tarwin	980419	**Australian fur seal** *Arctocephalus pusillus doriferus*	F	November 1997	145	98	58

### Foraging Mode and Feeding Cycle

To make direct observations of the foraging tactics used by captive fur seals, we presented each seal individually with dead prey items dropped down a 15 cm diameter PVC pipe positioned in front of the pool's underwater viewing window. This ensured that the seals would encounter the prey items free-floating in the water column approximately 1 meter underwater. Multiple prey items were dropped down the pipe at once to encourage the seals to capture and consume the prey items consecutively underwater during the dive. If the prey was found to float in the pipe or was sinking too slowly, a bucket of pool water was poured down the pipe to flush it out. Two species of fish were used in this trial: yellowtail scad (*Trachurus novaezelandiae*, mean fork length ± s.e.m: 152.9±2.15) and pilchard (*Sardinops* sp., mean fork length ± s.e.m: 144.5±2.71). Unfortunately it was not possible to use live prey during these trials.

We used a high-definition video camera (Sony NX70), filming at 50 frames/second, to record the feeding events through the pool's underwater viewing window. The camera's frame rate was used to measure the duration of some important components of the feeding cycle by counting the number of frames between text markers placed into the video footage using Adobe Premiere Pro CC (Adobe Systems Inc., San Jose, California). We measured the time duration from the first frame where the mouth is seen to open until the frame where maximum gape is achieved (duration of jaw opening). Maximum gape was measured as the distance between the tip of the upper and lower lips; this should be considered an estimate as variation in the orientation of the animal made it very difficult to make precise measurements. Gape was measured using ImageJ 1.45 s (National Institutes of Health, USA). We then measured the duration from maximum gape until the frame where the jaws were fully closed to grip the prey item between the seal's teeth (duration of jaw closing). After the initial capture, prey was often manipulated during further jaw movements until it was transported fully into the mouth and swallowed. The number of mouth movements or chews was tallied and the duration was measured from the last frame of the first jaw closure until the last frame of the final jaw closure that marked the end of the feeding event (duration of prey manipulation). The total duration of the feeding event was measured from the beginning of jaw opening until the end of prey manipulation (event duration). Due to variation in the orientation of the animal it was only possible to make these measurements in a subset of feeding events filmed. Only 11 out of 49 events filmed for Tarwin, and 20 out of 45 for Bay, were included in this analysis.

### Variation in Feeding Behaviours

To explore the range of foraging behaviours used in different scenarios, we presented each seal with three different methods of prey presentation. In each method we presented the seal with six dead fish (three whiting *Sillago* sp. and three pilchards *Sardinops* sp.), drawn from their regular daily diet. Again, feeding trials were performed individually for each seal to prevent competition between animals. In the first method we threw the six prey items loosely around the surface of the pool so that the seal encountered them free-floating in open water (scatter feed). For the second presentation method we placed the six fish into a hollow plastic ball with small, round openings in its side. The ball was attached to an elastic bungee cord that allowed it to be manipulated and pushed around in the water by the seals (mobile ball device).

In the third method, prey were concealed within a static feeding device that could not be manipulated to knock out the hidden prey (static box device). The device was made from a plastic storage box to which a plastic front-plate had been attached into which we had set recessed PVC tubes. These tubes were arranged in four columns that alternated between 5 cm and 10 cm diameter tubes. The top and bottom tubes were connected on the inside of the device, while the middle tube opened into the internal cavity of the box. To prevent fish falling into the connecting tube or the inside of the box, fiberglass fly-wire mesh was glued to the inside ends of the recessed tubes. The box was attached to a solid wooden frame that was temporarily tied to the pool fence with Velcro straps. The box could be raised or lowered on wooden beams that slid over the frame, before being locked into a static position either above or below the water. When in use, the six fish were placed into the recessed tubes and a separate plastic board was hooked over the front of the device. The box was then lowered into the water and locked in position, before the plastic board was pulled away. This board prevented fish falling out of the device with the flow of bubbles as it was lowered into the water.

To conduct these feeding trials one seal was given access to the main pool and left for 10 minutes to acclimate before the experimental session began. After acclimation, one of the seal's keepers entered the exhibit to present it with its prey using one of these three methods. We then filmed the seal's behaviour at 25 frames/second from above and below the water for a 20-minute observation period to document all of the behaviours associated with capturing and handling their food. After the first seal completed its experimental session the seals were swapped so that the second seal could participate. Each seal only participated in one experimental session per day and all sessions were carried out between 0730–1030 h before their first training session. We carried out five replicates of each experimental treatment for each seal, so in total the two Australian fur seals were presented with 30 prey items for each of the three prey presentation methods.

The video footage for each experimental session was viewed in Adobe Premiere Pro CC so that the behaviours used to capture and handle prey could be compared among treatments. Where visible, the initial prey capture tactic for each prey item was recorded as either biting or suction. The frequency of these was compared among the three prey presentation methods for each seal using a Pearson's chi-squared test using the standardized residuals to assess significance based on the critical value of ±1.96, which corresponds to an alpha (α) of 0.05. Statistical analyses were conducted using R statistical and graphical environment (R version 3.0.2, R development core team, 2013) [17].

## Results

### Foraging Mode and Feeding Cycle

**Figure 1:**
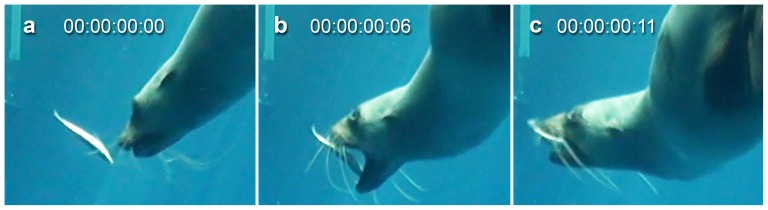
Raptorial biting used to capture a prey item floating in open water filmed at 50 frames/second. a) Jaws are closed as the seal approaches the prey item, b) Jaws open to maximum gape and overtake the prey item, c) jaws snap shut over prey item capturing it between the postcanine teeth. Prey capture event performed by Bay (ARKS# A70598), a female Australian fur seal (*Arctocephalus pusillus doriferus*). Time displayed as hours:minutes:seconds:frames. For the footage see Video S1.

**Figure 2:**
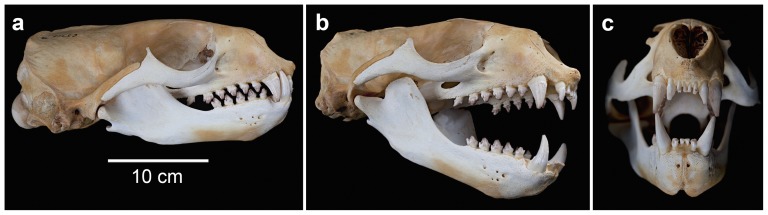
Australian fur seal skull and dentition (*Arctocephalus pusillus doriferus*; NMV C5717). a) jaws in occlusion showing simple interlocking postcanines, b) jaws opened to approximate gape used during raptorial biting, c) circular opening at front of the mouth formed by the canines and incisors when gape is small.

**Figure 3:**
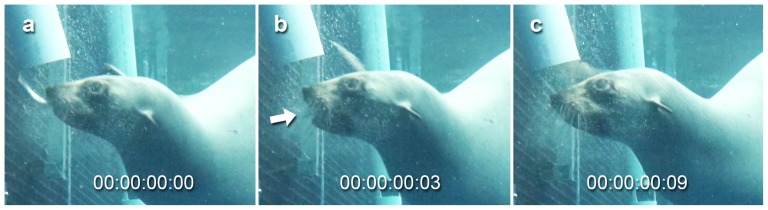
Suction used to draw a prey item into the oral cavity filmed at 50 frames/second. a) prey item held loosely between the lips, b) prey item sucked into the oral cavity as the jaws opened to maximum gape, the arrow indicates the direction of prey movement towards the mouth, c) mouth closes and water is expelled via the lateral sides of the mouth, although this is difficult to see in cloudy water. Prey capture event performed by Bay (ARKS# A70598), a female Australian fur seal (*Arctocephalus pusillus doriferus*). Time displayed as hours:minutes:seconds:frames. For the footage see Video S1.

When capturing free-floating prey in open water both subjects primarily used raptorial biting to secure their prey (Fig. 1; Video S1). This involved opening and snapping their jaws shut over the prey item so that it was caught between the teeth (Fig. 2). Most prey items were captured from the side so that they were held between the postcanine teeth at the end of the first bite (Fig. 1c). In many events suction was used in combination with biting to draw prey to within range of the teeth as part of the initial capture (Video S1). This was identified through movement of the prey item towards the mouth before the jaws snapped shut. Following the use of suction, lateral water expulsion was often visible, with water that had been drawn into the mouth during suction being purged via the sides of the mouth. However, in some events the prey item showed no movement towards the mouth and no water expulsion was visible after the initial bite, indicating that little or no suction was used in these events. If only a small amount of water was drawn into the mouth it is possible that this could have been swallowed along with the prey rather than being expelled, or expelled without being visible. In a small number of events extremely rapid suction appeared to play a major role in drawing prey almost fully into the mouth before the jaws closed (Fig. 3; Video S1). This occurred when the seal approached the prey item head on rather than from the side, possibly because the item could be more easily drawn directly into the oral cavity when in this orientation, rather than being first caught laterally between the postcanine teeth.

Once captured between the teeth, further mouth movements or chews were used to re-orient the prey item before it was drawn fully into the oral cavity (Video S1). Suction was likely used to transport the prey item from where it was held by the teeth into the oral cavity, as indicated by the performance of lateral-water expulsion following transport of the prey item. Unfortunately it was not possible to identify when swallowing occurred within the feeding cycle. Kinematic variables are summarized in Table 2.

**Table 2:** Summary of kinematic variables.

Kinematic Variable	Bay	*N*	Tarwin	*N*
Jaw opening (sec)	0.095±0.006	20	0.093±0.006	11
Jaw closing (sec)	0.068±0.005	20	0.082±0.011	11
Prey manipulation (sec)	1.125±0.116	20	1.980±0.172	11
Event duration (sec)	1.288±0.116	20	2.155±0.169	11
Maximum gape (cm)	6.753±0.341	20	5.600±0.342	11
Number of Jaw movements	4.800±0.485	20	6.818±0.352	11

Values are means ± s.e.m.

### Variation in Feeding Behaviours

Both subjects varied their foraging behaviours for the three methods of prey presentation. When targeting prey during the scatter feed treatments, they swam rapidly as they approached and captured each of the six prey items near the surface soon after they landed in the water. Both seals used raptorial biting as the primary prey capture tactic in 100% of feeding events where the initial capture tactic was clearly visible. However, although raptorial biting was considered the primarily capture tactic used, it is likely that some suction was also used in combination with biting to draw prey within range of the teeth, as observed in the first part of this study. Most prey items were captured in the jaws by biting near the head of the fish so that the postcanine teeth pierced the prey item. If first captured near the tail or from the side, the prey item was manipulated underwater using mouth movements or chews to re-orient it before it was swallowed head first.

**Figure 4:**
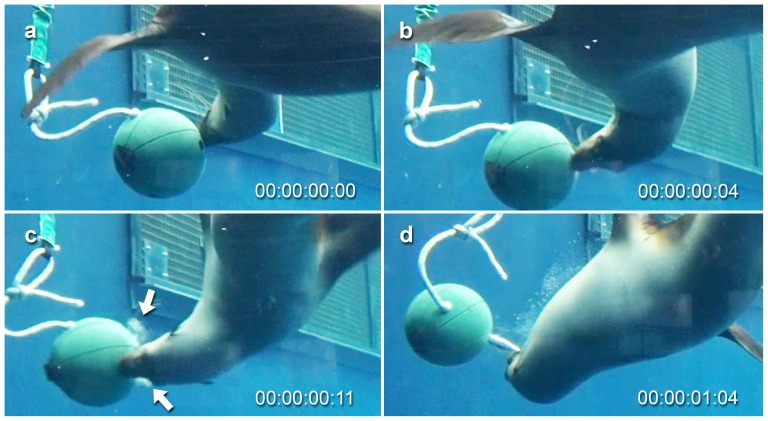
Object manipulation and suction used to draw prey from the mobile ball device filmed at 25 frames/second. a) ball was carefully manipulated using the muzzle, while looking through the hole in its side for concealed prey, b) when a prey item is seen near the opening the seal moved its mouth over the hole before pushing the ball forward, while also generating suction to draw out the prey item, c) water expulsion visible as it is expelled via the sides of the mouth following suction (arrows indicate cloud of turbid water being expelled), d) once protruding from the hole the prey item was gripped using the anterior teeth before being pulled out and consumed. Prey capture event performed by Bay (ARKS# A70598), a female Australian fur seal (*Arctocephalus pusillus doriferus*). Time displayed as hours:minutes:seconds:frames. For the footage see Video S1.

We found both seals to manipulate the mobile ball device by pushing it with their muzzles in an effort to knock out concealed prey items (Fig. 4). Bay in particular would carefully manipulate the ball with her muzzle, while looking through the hole in its side to see where the prey items were located (Fig. 4a). When a fish floated close to the opening, she moved her mouth over the hole and rapidly pushed the ball forward to knock the fish into her mouth (Video S1). If a fish started to fall from the ball it was gripped by biting with the anterior teeth, before being pulled from the device and consumed. If the prey item remained concealed within the ball it was extracted using suction. This involved the seal positioning its mouth over the opening in the ball (possibly using the vibrissae to locate the opening) and pushing it, while also generating suction to draw out the fish. Use of suction was indicated by lateral water expulsion, where water drawn into the mouth was expelled via the sides of the mouth after the initial capture (Fig. 4c). When using this device Bay used biting as the initial capture tactic in 64.3% of feeding events, while suction feeding was used in 35.7%. Tarwin was similar with 68.8% of prey initially captured by biting, while 31.3% was captured by suction.

**Figure 5:**
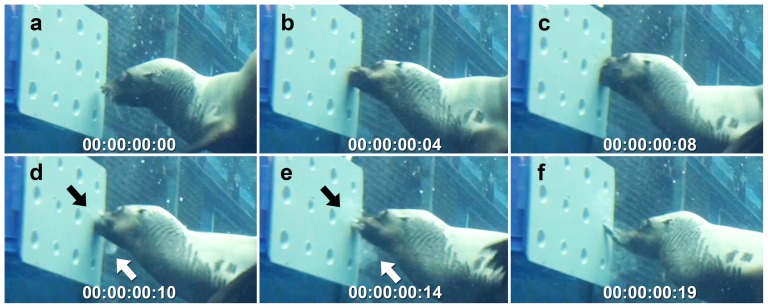
Suction used to draw prey out of the static box device filmed at 25 frames/second. a) prey item was found by carefully looking into each recessed tube, b) mouth was positioned over the opening using eyes and whiskers, c) suction generated by widening the gape and retracting tongue and hyoid, d) jaws closed to grip prey with anterior teeth, while performing lateral water expulsion where seawater drawn into mouth during suction was expelled via sides of the mouth (arrows indicate cloud of turbid water being expelled), e-f) pull prey out of device while holding it with the anterior teeth before performing further manipulation and swallowing. Prey capture event performed by Tarwin (ARKS# 980419), a female Australian fur seal (*Arctocephalus pusillus doriferus*). Time displayed as hours:minutes:seconds:frames. For the footage see Video S1.

Both seals also used suction when capturing prey from the static box device. They explored each tube in turn with their eyes and vibrissae until they located a hidden prey item. If a prey item floated partially out of the device it was captured by biting, using the anterior teeth, before being pulled out of the device and consumed. If fully concealed, the seal placed its mouth over the targeted recessed tube and sucked the prey item out (Fig. 5; Video S1). Water that had been drawn into the mouth along with prey item was purged via lateral water expulsion as the jaws closed over the fish (Fig. 5d). For both subjects, suction feeding was used as the main prey capture tactic when drawing prey from the static box device. Bay used suction feeding in 67.9% of feeding events while Tarwin used it in 79.3% of feeding events. Remaining prey captures were performed using biting after the prey item had floated partly out of the device and to within range of the teeth.

**Figure 6:**
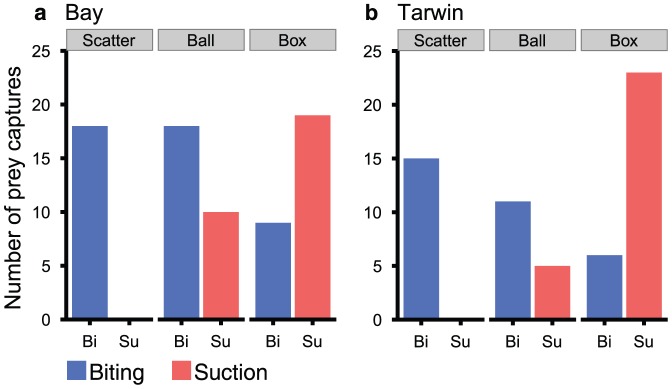
Biting versus suction as initial prey capture tactic when encountering prey in different ways. Number events where biting (Bi) or suction (Su) was the initial prey capture tactic when prey was presented as part of a scatter feed or when using the mobile ball or static box feeding device. Data were recorded for each prey item except where the capture tactic was unclear from the video playback.

We found that prey presentation method had a significant effect on whether prey was initially captured by raptorial biting or suction for both subjects (Bay: χ^2^ (2) = 21.4, p<0.01, Tarwin: χ^2^ (2) = 27.07, p<0.01). Significantly more prey items were captured by biting and fewer by suction, during the scatter feed where no suction feeding events were observed (standardized residuals  =  Bay 3.92, Tarwin 4.18; Fig. 6). In contrast, the opposite was true when capturing prey from the static box device, where both seals used significantly more suction (and significantly less biting) to draw out the concealed prey items (standardized residuals  =  Bay 3.94, Tarwin 4.9; Fig. 6). While raptorial biting was used more frequently than suction when using the mobile ball device, this difference was not found to be statistically significant for either seal (standardized residuals  =  Bay 0.48, Tarwin 1.44; Fig. 6).

In addition to suction and biting, both subjects were also observed to blow bubbles out of their noses towards or into the mobile ball and static box devices. When using the mobile ball device, bubbles were blown either below the ball or into the hole in its side. As they flowed around and through the device they may have helped to knock out concealed prey items. When using the static box device Bay seldom used bubbles and blew them in the direction of the device without targeting a specific prey item or recessed tube. Tarwin in contrast appeared to use bubble blowing as a targeted foraging tactic where she pressed her nose against the tube and blew a focused stream of bubbles directly into it at the hidden prey item (Video S1). This behaviour was used in all of Tarwin's static box sessions; however, it was generally only used after most of the prey items had already been captured by suction and so only one prey item was successfully captured using this tactic.

It is interesting to note that regardless of the enrichment treatment or prey capture tactic used, the prey items caught in this study were almost all swallowed head first. It was possible to identify whether the prey were swallowed head or tail first in 88 of the 155 fish fed out and of these, 85 were eaten head first, while only three were eaten tail first. Some of the remaining prey items (that could not be distinguished as having been either swallowed head or tail first) were processed by shaking at the surface before being swallowed. This involved the seal holding the prey item in its postcanine teeth before vigorously shaking its head from side to side until the prey item ripped in two (Video S1). When it broke, half the prey item was thrown across the pool while the other half was retained in the mouth and swallowed. The seal then collected and consumed the remaining pieces from around the pool.

## Discussion

These results show that while fur seals do indeed use raptorial biting as their primary prey capture mode when targeting prey in open water, in other scenarios suction feeding is more frequently used where it allows them to capture prey that cannot be caught by biting alone. Even when performing classic raptorial biting, suction was often still used in combination with biting to draw prey to within range of the teeth. This may be very important during wild feeding when pursuing evasive prey, as suction generation would counteract any water flow away from the seal's mouth as a product of the compressive bow wave generated by the seal's movement through the water [18].

As we would expect, the phases of the feeding cycle when performing raptorial biting were found to differ from those identified in the feeding cycle of primarily suction feeding marine mammals [5], [7], [19]–[21]. No preparatory phase was identified where the jaws were opened to a partial gape (10–30% of maximum gap) prior to the jaw opening fully. This type of preparatory phase has been identified in a number of species of suction feeding odontocete cetaceans [19]–[21] as well as in bearded seals [5]. In this aspect Australian fur seals are similar to leopard seals and harbour seals, which have also been found to lack a distinct preparatory phase prior to the main jaw opening [6], [7].

Jaw opening was similar to that observed in other marine mammal species, but rather than the prey item being drawn directly into the oral cavity during the subsequent gular or hyolingual depression phase, where suction is generated by retraction of the tongue and hyoid apparatus, the jaws were instead snapped shut on the prey item so that it was caught between the teeth. Once captured, the prey item was manipulated with further mouth movements or chews, before it was drawn fully into the oral cavity for swallowing. The use of suction for transporting the prey item from where it was held by the teeth into the oral cavity was very similar to that described for captive leopard seals [6].

When targeting prey in open water the fur seals consistently aimed for the fish's head. This presumably allows the seal to more easily swallow the prey head first, minimizing risk to the seal of being pierced by the fish's spines as it passes down the seal's throat. When pursuing a fleeing prey item in the wild, the fish's head would presumably also be the body part furthest away from the seal's mouth. Therefore it is possible that by initially aiming for the head, seals are more likely to capture the prey item, even if they hit it further down the body. Targeting the head might also function to kill or disable prey more quickly, allowing the seal to subsequently consume it using as little energy as possible.

At small gapes, the canines and incisors of an Australian fur seal form a circular mouth opening (Fig. 2c). It is possible that this formation assists with the generation of focused suction at the front of the mouth when the gape is small (Fig. 3b). Formation of a circular mouth opening has been found to be important in other suction feeding pinnipeds, including bearded seals [5], harbour seals [7] and walruses (*Odobenus rosmarus*) [22], [23]. Leopard seals were found to seal off the lateral sides of their mouth using their cheeks when performing suction feeding [6]. The fur seals in this study likely used their cheeks in a similar way, especially when using suction alone or when using it in combination with raptorial biting (Video S1).

The preference shown by Australian fur seals for using suction feeding when drawing prey from the static box device is similar to observations in other pinniped species. In captive feeding trials, bearded seals were found to only use suction when capturing prey, even when that prey protruded from the surface of a static feeding apparatus so that it was within range of the teeth [5]. When using a feeding apparatus, harbour seals were found to use suction feeding in 84% of feeding events, with remaining prey items captured by biting, before being drawn out and consumed in a similar manner to what we observed in captive Australian fur seals [7]. In contrast, when capturing live, free-swimming prey harbour seals were found to use raptorial biting to capture large prey, while small prey was captured by suction [24]. When we observed our subjects to use rapid suction alone to capture prey in open water, it was to capture the smaller pilchards, suggesting that Australian fur seals may also show a preference towards suction feeding when capturing smaller prey. In these events the prey items were captured from directly in front of the mouth. This is similar to what was observed in captive leopard seals by Hocking et al. [6] where prey was sucked into the mouth from within approximately 5 cm of the tip of the muzzle. In contrast, crabeater seals were observed to suck pilchards out of a channel (section of pipe cut in half) from up to 50 cm [4].

In both subjects the eyes remained open throughout the prey capture event as seen in Figs 1-5. This contrasts with observations in phocids, where bearded seals [5], harbour seals [7] and leopard seals [6] were found to close their eyes during the initial capture. Maintaining visual contact throughout the prey capture event may assist when targeting fast or evasive prey during daytime epipelagic foraging. When targeting benthic prey in association with the seafloor we might expect Australian fur seals to close their eyes to protect them against substrates that might be abrasive to the eyes [5]. There is also less light in deep water and Australian fur seals are known to forage both during daylight hours and at night [16]. Marshall et al. [5] suggested that bearded seals might close their eyes to increase the tactile sensory modality, by closing down their visual sense. Both subjects were observed to use their vibrissae as well as their vision to locate their prey; however, it is possible that maintaining vision during prey capture is more important in Australian fur seals than it is in these other pinnipeds. Alternatively, there may simply be less risk to the eyes during wild foraging in Australian fur seals, lessening the need to close their eyes during the initial capture, although this seems less likely given their benthic foraging habits.

Following use of suction, lateral water expulsion was observed. This likely allows the seals to swallow prey without ingesting large quantities of seawater. It also functions to re-set the feeding apparatus by emptying it of seawater in preparation for the next suck. In these trials water expulsion was clearly observed as a cloud of bubbles and turbid water being ejected from the lateral sides of the mouth. This was very similar to that described in captive leopard seals [6] and harbour seals [7]. It is possible that some water expulsion could occur without suction, as it is likely that a small volume of water would be captured in the mouth even during raptorial feeding; however, strong lateral water expulsion of a larger volume was not observed in the absence of suction. The presence of clear lateral water expulsion seems to be a good indicator of whether suction was used during the prey capture. When water expulsion is forcefully directed out of the front of the mouth, rather than laterally via the cheeks and postcanine tooth row, it is known as hydraulic jetting and can be considered “the opposite behaviour to suction” [5]. This behaviour has been recorded in a number of pinniped species including bearded seals [5] and harbour seals [7] where it has been used to knock concealed prey out of feeding apparatuses. In these studies it was detected as a positive spike in ambient water pressure. It is possible that the Australian fur seals used in this study may also have used hydraulic jetting when knocking prey from concealment within the static box device. Lateral water expulsion was clearly visible in alternation with suction as water was expelled via the sides of the mouth in preparation for the next suction event. However, it is possible that some water was also directed anteriorly into the recessed tube, where it may have assisted in dislodging the prey item before it was drawn out with the next suck. Unfortunately, we were not able to make direct measurements of the ambient water pressure within the recessed tubes as part of this trial. Hydraulic jetting is used by walruses and bearded seals to help excavate benthic infauna from sediment during wild foraging [5], [22], [23]. Harbour seals have also been observed in the wild digging in soft sediment with their flippers and muzzle when hunting sand lance (*Ammodytes dubius*) [25]. Given the benthic foraging habits of the Australian fur seal, it is possible that they too use suction feeding and hydraulic jetting when targeting cryptic prey.

Another behaviour that may be useful when targeting cryptic prey is the use of focused bubble blowing to knock or scare prey out of hiding. This type of bubble blowing has previously only been observed in wild Weddell seals (*Leptonychotes weddelli*), which having been found to blow bubbles into crevices in the ice to flush out fish so that they could be captured in open water [26]. Given that only one of our two subjects displayed targeted bubble blowing as a foraging tactic, it is possible that this individual learned this behaviour in captivity. But given the benthic foraging habits of this species, it is still possible that Australian fur seals hunting near the seafloor use this type of behaviour.

It is unclear why some of the prey items were taken to the surface to be processed by shaking prior to being swallowed in pieces. All of the items used in this study were small fish of a similar size, so that we could determine if prey presentation method, rather than the properties of the prey item, altered their foraging behaviours. In the wild, Australian fur seals have been observed to process prey that is too large to swallow whole using shake processing. Given that all of the prey items presented in this study were of a size that could easily be swallowed whole, it seems likely that the prey items were processed by shaking simply as a form of play behaviour. We are further exploring the use of prey processing when handling different types and sizes of prey as part of future research.

The results of these captive trials confirm the hypothesis that Australian fur seals primarily use raptorial biting as their default feeding mechanism when capturing free-floating prey; however, rather than simply involving snapping at prey with the jaws, this study showed that raptorial feeding is instead a complex foraging behaviour that involves the combined use of biting and suction to efficiently capture prey. Given that these seals are also able to use strong suction alone to draw prey into the oral cavity, we must conclude that rather than being a less efficient prey capture strategy [3], raptorial biting must be adaptive for otariid seals that have evolved to favour this feeding mode. The seals in this study also displayed great flexibility in their foraging behaviours, with the ability to employ a range of other tactics, including suction feeding, bubble blowing and possibly hydraulic jetting, when encountering prey under different conditions. While raptorial biting was the default tactic used when hunting free-floating prey, focused suction feeding was the most common tactic used when uncovering or extracting hidden prey. Given the similarity in morphology between the different species of otariid seals, it is likely that many others perform an equally broad range of behaviours when hunting at sea. For the Australian fur seal, this combination of behaviours is likely important to their success as top predators, allowing them to successfully exploit a huge diversity of prey species in environments ranging from the seafloor to the water's surface.

## Supporting Information

Video S1 **Prey capture and handling behaviour in Australian fur seals (** ***Arctocephalus pusillus doriferus*** **).**
(MOV)
